# Development of the Social Housing of Ontario (SHO) Registry by health region: a platform for health research with the social housing population

**DOI:** 10.3389/fpubh.2026.1770282

**Published:** 2026-03-06

**Authors:** Gina Agarwal, Melissa Pirrie, Mikayla Plishka, Kumindu Gamage, Ricardo Angeles, Jasdeep Brar, Christie Koester, Guneet Mahal, Francine Marzanek, Manasvi Vanama

**Affiliations:** 1 Department of Family Medicine, McMaster University, Hamilton, ON, Canada; 2 Health Research Methods, Evidence, and Impact McMaster University, Hamilton, ON, Canada

**Keywords:** administrative data, health equity, low-income naturally occurring retirement communities, registry, seniors, social housing

## Abstract

**Introduction:**

Social housing plays a critical role in addressing housing inequality and promoting well-being. This paper examines the creation of a registry of social housing sites across all six Ontario Health (OH) regions.

**Methods:**

For all 47 housing service providers in Ontario, social housing address, provider type, and building type were extracted from their website or from documents provided by the housing organization. The Registry included rent-geared-to-income housing with unique or minimally shared postal codes. Descriptive statistics were analyzed for the housing site characteristics aggregated by OH region.

**Results:**

2,109 social housing sites were included in the final Registry, including 472 designated as seniors only (low-income naturally occurring retirement communities, LI-NORCs). There were regional differences in the proportions of each tenant designation, postal code uniqueness, housing provider types, and building classifications. For instance, the Toronto and North West regions had higher government-owned social housing sites (61% and 57%), whereas the East region had more non-profit owned sites (55%). Apartments were the most common building type across regions (57%), with varying proportions of townhouses and single/semi-detached houses.

**Discussion:**

A social housing registry, the SHO, has been established, serving as a valuable resource for health research, especially for marginalized populations. It can be linked to other datasets for future studies. The SHO Registry provides a robust method for the determination of LI-NORCs (low-income naturally occurring retirement communities).

## 1 Introduction

Vulnerable individuals, such as those with low socioeconomic status, older adults, single-parent households, and individuals with a history of homelessness, frequently depend on affordable housing options for their residence (1, 2). Providing subsidized housing is a common strategy implemented to address homelessness, poverty, and housing inequality that disproportionately impacts these vulnerable groups. In 2017, Canada introduced the National Housing Strategy, which focuses on providing affordable housing, where the rent is priced at or below market rent for low-to-moderate-income households (3, 4). Similarly, social housing is a government-assisted type of subsidized housing where the rent is typically capped at 30% of household income (5). These dwellings are usually owned by service managers, non-profit organizations, or co-operatives and managed by municipal or district service managers (6). As of 2021, Statistics Canada reports that 842,000 Canadians aged 15 years or older were being provided with subsidized housing (7). In Ontario, there are an estimated 270,000 social housing units managed by 47 municipal or district service managers, demonstrating a significant resource commitment to this housing strategy (8).

This vulnerable population of social housing residents has low health literacy and often experiences barriers to accessing primary and preventative healthcare and poor quality of life (9). In addition, approximately 28% of social housing residents are seniors, with 7% of residents being over the age of 80 compared to 4% in the general population (10). As a result, high rates of chronic health conditions, such as cardiovascular diseases (22.70%), diabetes (10.54%), hypertension (59.12%), high cholesterol (43.59%), and higher rates of mortality are reported in this population (11). The higher prevalence of chronic diseases and mortality that affect social housing residents have been associated with the housing type and location (12, 13). There is an urgent need to address these rising health issues in this population, which adversely impacts their quality of life and places excessive burden on the healthcare and emergency response systems (12).

In order to identify priority health concerns of individuals residing in social housing, and to be able to specifically target low-income seniors living in naturally occurring retirement communities, which we have termed LI-NORCs, it is necessary to create a database or registry of social housing sites. However, there remains a lack of consistency in the information available and accessibility of the information from the 47 municipal and district social housing providers in Ontario. Furthermore, the lack of variables within health administrative data to identify individuals as social housing residents makes it difficult to examine potential associations between resident health issues and social housing conditions. We propose to address this gap in available resources by developing a registry of social housing sites that currently services populations across Ontario and can be linked to administrative data. The aim of this study was to create a registry, called the Social Housing of Ontario (SHO) Registry, comprising all social housing sites in Ontario that can be identified using administrative data.

## 2 Methodology

### 2.1 Site identification

We identified the 47 municipal and district housing providers from the Government of Ontario website (14). Social housing addresses were collected either directly from each housing provider's website or from publicly available housing application materials, and housing providers were contacted if this information was not available online. Written permission from the housing providers was obtained when required by the terms and conditions of the websites.

For each municipal/district housing provider, the data collected for each available housing site included: street address, postal code (if available), number of units, specific housing program (e.g., rent-geared-to-income, supplement, other special programs), tenant eligibility (e.g., senior, family, singles), housing provider type (non-profit organization, government-owned or a cooperative), building type (e.g., detached house, apartment building), and other building characteristics (e.g., number of stories, elevator availability, common room space). Any information that was not available was entered as “not specified.”

### 2.2 Extraction and classification of postal codes

For each housing site, its postal code or postal codes were determined using the Canada Post “Find a Postal Code” web-based tool (15). Disagreements between the postal codes provided by the housing provider and Canada Post were resolved using Google Maps and by consulting the housing provider when needed. The most common scenario was that a social housing site was large enough to have multiple postal codes, but only one was listed in the housing provider's materials. The second most common situation was a typo in the social housing provider's materials. In one instance, the housing provider listed a non-existent postal code (per Canada Post) that was nonetheless used on health cards by some residents (according to OHIP data when linked with administrative data) (16), so both postal codes were retained in the Registry. For the purposes of this Registry, social housing sites that shared a postal code were combined and considered one social housing site.

Next, all postal codes were entered into the same Canada Post tool to conduct a reverse look-up and identify all other residences or businesses sharing the same postal code. Where there were no other residences or businesses sharing the same postal code as the social housing site, the postal code was categorized as “unique” to social housing; in other words, 100% of that postal code catchment area was social housing units. This was further validated by visualizing the site on Google Maps, identifying the addresses of all adjacent buildings, and entering those addresses into the Canada Post tool.

For postal codes that *were* shared with neighboring addresses, an additional step was taken to calculate the proportion of social housing units within each postal code catchment area. For example, if a social housing apartment building with 60 units shared a postal code with 10 detached houses that were not social housing, then that postal code catchment area was 86% social housing units. Postal codes were categorized as “minimally shared” if social housing represented 50% or more of the units within its catchment area. Postal codes with less than 50% of units being social housing were categorized as “not unique or minimally shared.” For data verification, this full process was repeated by a second research staff member to reduce the likelihood of data entry errors in the final Registry.

### 2.3 Eligibility criteria for registry inclusion

To be eligible for inclusion in the Registry, the social housing site needed to have rent-geared-to-income (RGI) units and have at least one postal code that was either “unique” (100% social housing) or “minimally shared” (≥50% social housing). Since the purpose of the Registry was to link to existing datasets (e.g., administrative health, census, built environment), sites were required to have at least 10 units and be linked to a Dissemination Area using Statistics Canada's Postal Code^OM^ Conversion File Plus (PCCF+) (17). In contrast, social housing locations were excluded if the housing provider used a rent supplement form of payment, as these are typically tied to the individual and not the specific address where they currently reside. Other special programs for specific populations (e.g., Indigenous housing programs, supportive housing) were also excluded.

### 2.4 Site measures

The data was collated, and a unique site ID was assigned to each social housing location. For housing locations with multiple addresses (e.g., a cluster of townhouses), the same site ID was assigned to all addresses at that location. For each site ID, the following characteristics were compiled:

- Tenant designation: Housing providers designated some housing sites for specific tenant populations, such as seniors, families, or adults only. Where this information was not provided, the site was classified as “unspecified.” The sites designated for seniors are LI-NORC locations.
- Postal code type: Using the postal code classification process described in the methods above, each postal code was determined to be “unique,” “minimally shared,” or “not unique.” Since sites could have multiple postal codes, each site was then classified as having “unique postal codes,” “minimally shared postal codes,” or “mixed postal codes.” Sites where all postal codes were “not unique” were excluded from the Registry (see eligibility criteria).
- Provider type: Sites were identified as being owned by a private non-profit housing provider, government-owned, a co-operative, or mixed. The sites that did not provide this information were classified as unspecified.
- Building type: The building sites included in the Registry were classified as either an apartment, townhouse, single/semi-detached house, or a mix of these three types. Building type was classified as other/unspecified if the site did not provide information on building type or was of another type not included in the Registry.

### 2.5 Analysis

Descriptive statistics were used to describe the distributions of different characteristics of the housing sites included in the final Registry. The type of postal code, type of housing provider, and type of buildings available at every site were described for each district/regional housing provider. The descriptives were subsequently collapsed into the six Ontario Health Regions (West, East, Toronto, Central, North East, North West) to examine geographic distributions across the province and analyzed using chi-square and Cramer's V to test the statistical significance and effect size of the association, respectively. All analysis was completed using IBM SPSS Version 30.0.

## 3 Results

A total of 2,109 social housing sites met the eligibility criteria and were included in the final Registry. When aggregated by the Ontario Health regions (see Table 1), the region with the largest number of sites was the West region (*n* = 689), constituting 32.7% of the Registry, and the smallest was the North West region (*n* = 37) at 1.8%. All regions had LI-NORC sites designated for senior tenants only, ranging from 15.5% to 37.8% of their sites, for a total of 472 LI-NORC sites in the SHO Registry. This is a conservative count of LI-NORCs in the Registry since 478 sites (22.7%) were a mix of tenant types (e.g., a low-rise apartment building for seniors only and townhouses designated for families at the same site), and for 689 sites (32.7%), the tenant type was not specified by the housing provider.

**Table 1:** Total number of social housing sites and tenant types for the regions of Ontario.

**Ontario health region**	**Number of sites *N* (%)**	**Designated tenant type**				
		**Seniors** **(LI-NORC locations)** ***n*** **(%)**	**Families** ***n*** **(%)**	**Adults** ***n*** **(%)**	**Other/ Mixed** ***n*** **(%)**	**Not specified** ***n*** **(%)**
West	689 (32.7)	151 (21.9)	158 (22.9)	57 (8.3)	228 (33.1)	95 (13.8)
East	549 (26.0)	85 (15.5)	115 (20.9)	18 (3.3)	57 (10.4)	274 (49.9)
Toronto	439 (20.8)	114 (26.0)	0 (0.0)^a^	0 (0.0)^a^	9 (2.1)	316 (72.0)
Central	253 (12.0)	78 (30.8)	64 (25.3)	17 (6.7)	94 (37.2)	0 (0.0)
North East	142 (6.7)	30 (21.1)	32 (22.5)	1 (0.7)	75 (52.8)	4 (2.8)
North West	37 (1.8)	14 (37.8)	8 (21.6)	0 (0.0)	15 (40.5)	0 (0.0)
Total	2,109	472 (22.4)	377 (17.9)	93 (4.4)	478 (22.7)	689 (32.7)

^a^Toronto had a very high number of sites without a specified tenant type.

### 3.1 Postal code type

Across the six Ontario Health regions, nearly 75% of sites had a unique postal code, not shared with any other addresses (see Table 2). Toronto (82.2%) had the most sites with a unique postal code, but it was the most common finding across all regions (54.1% to 82.2%). The proportion of postal codes determined to be “minimally shared” was less than 25% for all sites except for the North West region (40.5%). Lastly, less than 5% of total social housing sites were classified as having a “mixed” postal code type where the site had at least one “unique” postal code and at least one “minimally shared” postal code. A small but statistically significant association between region and postal code type was found (χ^2^ = 74.8, df = 10, *p* < 0.00; Cramer's V = 0.13), indicating that postal code type was not evenly distributed across regions.

**Table 2:** Distribution of social housing sites based on type of postal code for regions of Ontario.

**Ontario health region**	**Total number of sites *N***	**Sites with unique postal codes *n* (%)**	**Sites with minimally shared postal codes *n* (%)**	**Sites with mixed postal codes *n* (%)**
West	689	477 (69.2)	179 (24.7)	42 (6.1)
East	549	376 (68.5)	161 (29.3)	12 (2.2)
Toronto	439	361 (82.2)	54 (12.3)	24 (5.5)
Central	253	204 (80.6)	48 (19.0)	1 (0.4)
North East	142	105 (73.9)	30 (21.1)	7 (4.9)
North West	37	20 (54.1)	15 (40.5)	2 (5.4)
Total	2,109	1,543 (73.2)	478 (22.7)	88 (4.2)

### 3.2 Housing provider type

In the Toronto and North West Ontario Health Regions, the majority of sites managed are government-owned housing providers (61.0% and 56.8%, respectively) (see Table 3). There was a statistically significant association observed between Ontario Health regions and housing provider type (χ^2^ = 195.0, df = 15, *p* < 0.001) with a moderate effect size (Cramer's V = 0.22), suggesting regional differences in the types of organizations providing housing. In the East Ontario Health Region, the majority of sites were owned by non-profit organizations (54.8%). The proportion of sites that were managed by a co-operative housing provider was consistently low amongst regions, with North West Ontario reporting the lowest at 0% and Toronto reporting the highest at 16.6%.

**Table 3:** Distribution of social housing sites based on type of housing provider for regions of Ontario.

**Ontario health region**	**Total number of sites *n***	**Type of housing provider**				
		**Private non-profit** ***n*** **(%)**	**Government** ***n*** **(%)**	**Co-operative** ***n*** **(%)**	**Mixed** ***n*** **(%)**	**Unspecified** ***n*** **(%)**
West	689	254 (36.9)	299 (43.4)	83 (12.0)	2 (0.3)	51 (7.4)
East	549	301 (54.8)	170 (31.0)	34 (6.2)	2 (0.4)	42 (7.7)
Toronto	439	98 (22.3)	268 (61.0)	73 (16.6)	0 (0.0)	0 (0.0)
Central	253	64 (25.3)	100 (39.5)	21 (8.3)	2 (0.8)	66 (26.1)
North East	142	52 (36.6)	44 (31.0)	14 (9.9)	1 (0.7)	31 (21.8)
North West	37	14 (37.8)	21 (56.8)	0 (0.0)	2 (5.4)	0 (0.0)
Total	2,109	783 (37.1)	902 (42.8)	225 (10.7)	9 (0.4)	190 (9.0)

### 3.3 Type of building

For all six Ontario Health regions, the most common building type for social housing sites was apartment buildings, followed by townhouses (see Table 4). Despite this similarity, using a Chi-square Test it was found that building type was not distributed evenly across the regions (χ^2^ = 341.6, df = 20, *p* < 0.001) and that this was a small-to-moderate association (Cramer's V = 0.20). The proportion of apartment buildings ranged from 35.1–83.4%, with Toronto having the highest proportion, followed by the Central region at 66.0%. In comparison, the West and East regions reported the highest proportions of townhouses (32.2% and 28.4%, respectively). For the remaining regions, the proportion of townhouses ranged from 2.7% to 20.2%. Single/Semi-detached housing was the least common building type across all regions, except for the North West (13.5%). In addition, a mixture of building types were seen for sites with more than one listed address, with all six regions reporting between 1.8% and 16.2% of sites being mixed. Finally, the proportion of sites that had building types other than the main three types or had no information on building type was the highest in the North West region at 32.4%.

**Table 4:** Distribution of social housing sites based on the type of housing building for regions of Ontario.

**Ontario health region**	**Total number of sites *n***	**Building type**				
		**Apartment** ***n*** **(%)**	**Townhouse** ***n*** **(%)**	**Single/ semi detached** ***n*** **(%)**	**Mixed** ***n*** **(%)**	**Other/ unspecified** ***n*** **(%)**
West	689	323 (46.9)	222 (32.2)	43 (6.2)	57 (8.3)	44 (6.4)
East	549	248 (45.2)	156 (28.4)	10 (1.8)	85 (15.5)	50 (9.1)
Toronto	439	366 (83.4)	62 (14.1)	3 (0.7)	8 (1.8)	0 (0.0)
Central	253	167 (66.0)	51 (20.2)	13 (5.1)	22 (8.7)	0 (0.0)
North East	142	86 (60.6)	21 (14.8)	10 (7.0)	20 (14.1)	5 (3.5)
North West	37	13 (35.1)	1 (2.7)	5 (13.5)	6 (16.2)	12 (32.4)
Total	2,109	1,203 (57.0)	513 (24.3)	84 (4.0)	198 (9.4)	111 (5.3)

## 4 Discussion

The SHO Registry of social housing sites across all six Ontario Health regions has been successfully established, including a valuable subset of LI-NORCs. This Registry will serve as a valuable resource for research on the social determinants of health and healthcare utilization, particularly focusing on vulnerable populations. It has been purposefully designed with the capability to be linked to other administrative datasets using postal code or census divisions (e.g., dissemination area). Importantly, the methodology employed to create this Registry is reproducible, enabling the refreshment of the Registry as the social housing landscape naturally changes and based on different reference dates as needed. In fact, since 472 social housing buildings are designated specifically as seniors' buildings, containing a high concentration of low-income seniors, we recommend that these sites be considered to be a subset of naturally occurring retirement communities (18), which we have named Low-income Naturally Occurring Retirement Communities or LI-NORCs. Due to their low income, these clusters of vulnerable seniors form specific groups of individuals for whom targeted health and social programs may be beneficial and more efficiently delivered. The SHO Registry provides a robust method for the determination of LI-NORC sites.

Moreover, the development of the SHO Registry addresses a critical gap by providing a centralized platform. Individuals seeking information about housing services in various regions and districts could collaborate with the research team and access this resource, eliminating the need for laborious searches or multiple application processes. This approach is especially beneficial for those with limited time or resources, such as housing providers with limited or no capacity for this type of analysis. Furthermore, the methodology used in creating this Registry can be extended to develop similar tools for other essential services, such as long-term care homes, food banks, and homeless shelters, which often lack comprehensive information resources.

When considering the implications of our research, it is essential to recognize how this methodology can be applied to social housing in other Canadian provinces/territories and in other countries. While there are over 350,000 social housing residents in Ontario (10), there are 842,000 across Canada (7). In the United States, public housing is priced significantly below the market rate, but it has faced challenges over time due to funding shortages and management issues (19). Similarly, council housing in the UK has historically been conducive to alleviating poverty, although recent trends indicate a decline in its availability and impact (20). The methodology used to create a registry of social housing sites in Ontario can be extended to create a centralized registry of public housing in the US and council housing in the UK. The establishment of such a registry would enable researchers to investigate the influence of housing on various health conditions, risk factors, quality of life, and social determinants of health in these countries.

Postal codes are pivotal in research, acting as markers to identify population areas, residential locations, as well as health conditions and risk factors (21–24). Researchers have employed diverse methodologies to establish comprehensive registries. A 2020 study in Ontario developed a screening method to determine the likelihood of identifying retirement home residents based solely on postal code information (25). This approach facilitates linkages to administrative databases when direct address-to-health linkage is unavailable. Our study similarly utilized postal code data and map-based technologies to identify the specific population of interest and construct a social housing registry. Both studies aim to enhance data availability for researching residents' sociodemographic profiles, healthcare needs, and health conditions over time. While Brath et al. (25) focused on algorithm development, our study utilized postal code information and geospatial tools to create a much-needed social housing registry in Ontario.

The statistically significant variations in the distribution of postal code, building types, and provider types across different regions can largely be attributed to the unique characteristics of each area (26, 27). Toronto, being the most densely populated geographical area in Ontario, has a higher proportion of sites with unique postal codes and social housing sites that consist of apartments. In contrast, the East Ontario Health region, characterized by low population density, exhibits a much higher proportion of social housing sites with unique postal codes and the second lowest proportion of apartment buildings among its building sites (28). Furthermore, Northern Ontario, covering nearly 90% of Ontario's land mass, has the lowest population density in the province. Interestingly, despite its sparse population, the North has the lowest proportion of social housing sites in the form of townhouses, along with a small number of detached or semi-detached houses. Due to elevated construction costs compared to Southern Ontario, there is limited availability of new housing (29). This can present significant challenges for new housing development, making it less financially viable to build detached or semi-detached houses compared to apartment buildings. Lastly, we recognize that there is a difference in the dominant type of housing provider between the regions. We can speculate that this stems from the decisions made by the individual municipalities regarding the allocation of funds. The diverse characteristics and unique challenges of each region in Ontario help shape the distribution and types of social housing across the province.

In our study, the primary limitation was the lack of information available on the websites and applications of some housing providers (e.g., tenant classification). This incomplete data collection process could impact the utility of the SHO Registry, as users may still need to seek out this information independently or may choose not to access housing data due to the lack of details. To address these limitations, subsequent revisions of the tool could explore alternative methods for gathering missing information (e.g., additional direct data requests to the housing providers). Another limitation was that the Dissemination Area for three sites could not be determined using Statistics Canada's Postal Code^OM^ Conversion File Plus (PCCF+) and therefore those sites needed to be excluded from the Registry; however, this was less than 0.2% of the sites.

## 5 Conclusion

To lay the groundwork for research on the health of social housing residents in Ontario, we have created a comprehensive social housing registry, further subdivided into a subset of LI-NORCs. This unique SHO Registry sets the stage for future research into the connections between resident health and social housing conditions across the six Ontario regions. Moving forward, developing an address-based tool could greatly enhance data collection and enable integration with other administrative databases, paving the way for more in-depth future studies.

## Data Availability

The data analyzed in this study is subject to the following licenses/restrictions: Available upon reasonable request. Requests to access these datasets should be directed to Gina Agarwal, gina.agarwal@gmail.com.
